# Dimensions of Compulsive Exercise across Eating Disorder Diagnostic Subtypes and the Validation of the Spanish Version of the Compulsive Exercise Test

**DOI:** 10.3389/fpsyg.2016.01852

**Published:** 2016-11-24

**Authors:** Sarah Sauchelli, Jon Arcelus, Roser Granero, Susana Jiménez-Murcia, Zaida Agüera, Amparo Del Pino-Gutiérrez, Fernando Fernández-Aranda

**Affiliations:** ^1^Department of Psychiatry, University Hospital of Bellvitge – Bellvitge Biomedical Research InstituteBarcelona, Spain; ^2^Centro de Investigación Biomédica en Red de la Fisiopatología Obesidad y Nutrición, Instituto de Salud Carlos IIIMadrid, Spain; ^3^Institute of Mental Health, Faculty of Medicine & Health Sciences, University of NottinghamNottingham, UK; ^4^Leicester Eating Disorder Service, Leicester Glenfield HospitalLeicester, UK; ^5^Department of Psychobiology and Methodology, Autonomous University of BarcelonaBarcelona, Spain; ^6^Department of Clinical Sciences, School of Medicine, University of BarcelonaBarcelona, Spain; ^7^Nursing Department of Mental Health, Public Health, Maternal and Child Health, The Nursing School of the University of BarcelonaBarcelona, Spain

**Keywords:** compulsive exercise, eating disorders, psychopathology, Compulsive Exercise Test, Spanish validation

## Abstract

**Objectives:** Compulsive exercise in eating disorders has been traditionally considered as a behavior that serves the purpose of weight/shape control. More recently, it has been postulated that there may be other factors that drive the compulsive need to exercise. This has led to the development of the Compulsive Exercise Test (CET); a self-reported questionnaire that aims to explore the cognitive-behavioral underpinnings of compulsive exercise from a multi-faceted perspective. The objectives of this study were threefold: (1) to validate the Spanish version of the CET; (2) to compare eating disorder diagnostic subtypes and a healthy control group in terms of the factors that drive compulsive exercise as defined by the CET; (3) to explore how the dimensions evaluated in the CET are associated with eating disorder symptoms and general psychopathology.

**Methods:** The CET was administered to a total of 157 patients with an eating disorder [40 anorexia nervosa, 56 bulimia nervosa (BN), and 61 eating disorder not-otherwise-specified (EDNOS)] and 128 healthy weight/eating controls. Patients were assessed via a semi-structured interview to reach a DSM-IV-TR diagnosis. Additionally, all participants completed the Symptom Checklist-90-Revised (SCL-90R) and the Eating Disorders Inventory-2 (EDI-2).

**Results:** Confirmatory factor analysis demonstrated adequate goodness-of-fit to the original five-factor model of the CET. BN and EDNOS patients scored higher in the avoidance and rule-driven behavior, weight control, and total CET scales in comparison to the healthy controls, and higher across all scales apart from the exercise rigidity scale compared to the anorexia nervosa patients. Mean scores of the anorexia nervosa patients did not differ to those of the control participants, except for the mood improvement scale where the anorexia nervosa patients obtained a lower mean score. Mean scores between the BN and EDNOS patients were equivalent. The CET scales avoidance and rule-driven behavior, weight of control and total CET scores were positively correlated with the clinical assessment measures of the SCL-90R and EDI-2.

**Conclusion:** Compulsive exercise is a multidimensional construct and the factors driving compulsive exercise differ according to the eating disorder diagnostic subtype. This should be taken into account when addressing compulsive exercise during the treatment of eating disorders.

## Introduction

Excessive exercise is a recurrent behavior in EDs, believed to be implicated in the etiology, development, and maintenance of these disorders (Davis et al., 1997; Taranis et al., 2011). Prevalence rates range between 34 and 55% (Dalle Grave et al., 2008; Bewell-Weiss and Carter, 2010; Sauchelli et al., 2015), and it has been associated with poorer treatment outcome (Shroff et al., 2006; Dalle Grave et al., 2008; Stiles-Shields et al., 2015), an increased likelihood of dropout during treatment (El Ghoch et al., 2013) and a shorter time to relapse (Strober et al., 1997; Carter et al., 2004). However, some studies have not found an association between exercise and treatment response (van Elburg et al., 2007; Kostrzewa et al., 2013), and there is evidence suggesting that supervised physical activity may be somewhat beneficial for outcome and attitudes toward exercise (Calogero and Pedrotty, 2004; Sauchelli et al., 2015).

The inconsistencies in the literature regarding the pathology of exercise in ED are likely to be due to discrepancies in the methodology of the studies. First, studies differ in the samples assessed; the duration of the disorder, age, and time point of assessment (baseline, lifetime prevalence, and during the treatment process). Second, an operationalized definition of excessive exercise is lacking. A quantitative approach to excessive exercise has been questioned; it is unclear whether focus should be placed on current or lifetime exercise or the amount/type of exercise required for it to be considered as excessive (Meyer and Taranis, 2011). It has, therefore, been argued that it is not the amount/intensity of exercise *per se* that is problematic in EDs, but the presence of a compulsive drive to engage in exercise (Meyer and Taranis, 2011; Meyer et al., 2011). Exercise can be considered as compulsive when the individual feels compelled to exercise, the behavior interferes with everyday life, relationships with others, and is continued despite potential harm to the self (Yates, 1991; APA, 1994). Compulsive exercise may be a more accurate representation of the pathological exercise profile of these patients given that it emphasizes the cognitions that maintain the behavior despite the damaging effects and that may hinder treatment progress. Yet, the majority of existing literature in adults with EDs has focused on the assessment of the total amount of exercise and the associated medical complications; only limited attention has been placed on its nature within these disorders.

It was originally believed that compulsive exercise in EDs was related to a need for body weight/shape control (Dalle Grave et al., 2008; Achamrah et al., 2016). More recent studies have suggested a multifactorial etiology (Meyer and Taranis, 2011; Meyer et al., 2011). Based on a cognitive behavioral model, Meyer et al. (2011) proposed four key constructs underlying compulsive exercising: (1) eating psychopathology; (2) obsessive compulsiveness; (3) mood regulation; and (4) perfectionism.

This multidimensional definition of compulsive exercise led to the development of a new measure, the CET (Taranis et al., 2011), which examines the emotional, cognitive, and behavioral characteristics of compulsive exercise from a multi-facet perspective. Further than exercising as a way to regulate mood and control body weight/shape, the instrument also evaluates the extent to which the maintenance of such unhealthy exercise behavior may be driven by the need to sustain a rigid schedule, to improve one’s mood despite a lack of enjoyment when exercising, or as a way to avoid negative emotions and feelings of guilt that may emerge when not exercising (Meyer et al., 2011; Taranis et al., 2011). In addition, it can be employed to distinguish a clinical from a non-clinical group (Meyer et al., 2016). The CET, with a five-factor structure, was first psychometrically tested in a non-clinical population (Taranis et al., 2011) and recently in a clinical setting as well (Meyer et al., 2016).

Meyer et al.’s (2011) model has been supported by several studies in both community (Goodwin et al., 2011, 2012, 2014a,b, 2016) and clinical adolescent groups (Formby et al., 2014; Noetel et al., 2016; Swenne, 2016). Fewer studies have examined a multidimensional function of exercise in adults with EDs. These studies, however, have found several associations between some of the dimensions described by Meyer et al. (2011), and greater exercise frequency, ED psychopathology, and clinical characteristics such as obsessions and compulsivity (Taranis and Meyer, 2010, 2011; Naylor et al., 2011; Taranis et al., 2011).

Despite the consensus on the relevance of exercise in the etiology of EDs, contradictory findings persist. Furthermore, thus far no study has examined the various factors (cognitive/emotional/behavioral) driving compulsive exercise, rather than actual exercise patterns, across ED diagnostic subtypes, and how each might be linked to ED symptoms and general psychopathology. The aims of this study were threefold: (1) to validate the Spanish version of the CET; (2) to compare ED diagnostic subtypes and a healthy control group in terms of the factors that drive compulsive exercise as defined by the CET; (3) to explore how the dimensions evaluated in the CET are associated with ED symptoms and general psychopathology. Based on the available literature we anticipated that the Spanish version of the CET would also allow us to differentiate between clinical and non-clinical groups, where participants with EDs would show elevated compulsive cognitions and behaviors toward exercise compared to healthy controls. We also hypothesized that greater compulsive cognitions would be associated with increased ED and general psychopathology.

## Materials and Methods

### Participants

The sample comprised 157 patients with EDs; 40 (25.5%) patients with AN, 56 (35.7%) patients with BN, and 61 (38.8%) patients with an EDNOS. The mean body mass indices of each group were 15.9 (*SD* = 1.6), 25.48 (*SD* = 6.6), and 22.97 (*SD* = 5.89), respectively. These patients were compared to 128 healthy weight/eating controls (mean body mass index = 22.06 *SD* = 2.54). A total of 228 (79.7%) participants were female (AN = 37; BN = 53; EDNOS = 53; controls = 85) and 57 (20.3%) were male (AN = 3; BN = 3; EDNOS = 8; and controls = 43). The ED groups were patients consecutively assessed at the ED unit of the University Hospital of Bellvitge, Spain. Diagnosis was made on the basis of the DSM-IV-TR (during the recruitment phase the DSM-5 had not yet been published), based on a semi-structured clinical interview (First et al., 1997). Controls were students from the local university (University of Barcelona, Spain). All participants had to be 18 years and over and speak Spanish at the level of a native speaker. In addition, the patients had to fully meet the diagnostic criteria. Potential control participants were excluded if they had a history of an ED, which was not the case. Mean ages were: 28.88 years (*SD* = 10.36) for the AN patients, 20.50 years (*SD* = 10.46) for the BN patients, 25.31 years (*SD* = 8.27) for the EDNOS patients, and 21.04 years (*SD* = 4.96) for the control participants.

### Measures

#### Compulsive Exercise Test (Taranis et al., 2011)

This self-reported questionnaire is designed to explore the emotional, cognitive and behavioral characteristics of compulsive exercise. It comprises 24 items answered on a 6-point Likert scale, from 0 (never true) to 5 (always true). Five subscales are derived: (1) avoidance and rule-driven behavior; (2) weight control exercise; (3) mood improvement; (4) lack of exercise enjoyment, and (5) exercise rigidity. Mean averages of each subscale are summed to obtain a CET total score. The psychometric properties of the CET have been validated in a non-clinical and clinical sample, showing high concurrent and convergent validity and Cronbach’s α ranging from 0.72 to 0.88 (Taranis et al., 2011; Meyer et al., 2016).

The translation into Spanish was conducted via the use of back-translation (Brislin, 1970). Two translators, experts in the field of EDs, provided a first translation from English to Spanish. A third examined the document for possible errors and resolved any doubts or inconsistencies. The Spanish version was then translated back into English, which was compared to the original English version. Throughout the process, language, grammar, and cultural discrepancies that might influence the interpretation of the questionnaire items were taken into account. Bilingual individuals overviewed the entire translation process.

#### Eating Disorder Inventory-2 (Garner, 1991)

This is a reliable self-reported questionnaire that explores typical cognitive and behavioral characteristics of ED. A total of 91 items, answered on a 6-point Likert scale, provide standardized scores on 11 subscales: drive for thinness, body dissatisfaction, bulimia, effectiveness, perfectionism, interpersonal distrust, interoceptive awareness, maturity fears, asceticism, impulse regulation, and social insecurity. The EDI-2 total score (sum of scores in each scale) was used to examine overall ED severity. Validation in a Spanish population (Garner, 1998) yielded a mean internal consistency of 0.63 (Cronbach’s α).

#### The Symptom Checklist-90-Revised (Derogatis, 1990)

This self-reported measure was used to assess general psychopathology. The 90-item questionnaire explores psychological problems and symptoms of psychopathology. A total of nine primary symptom dimensions are extracted: (1) somatization; (2) obsession–compulsion; (3) interpersonal sensitivity; (4) depression; (5) anxiety; (6) hostility; (7) phobic anxiety; (8) paranoid ideation; and (9) Psychoticism. Three global indices are also present: the Global Severity Index (GSI), designed to evaluate overall distress, the Positive Symptom Distress Index (PSDI), which indicates the intensity of the symptoms, and the positive symptom total (PST), which assesses self-reported symptoms. The test has been validated in a Spanish population (Derogatis, 2002), with a mean internal consistency of 0.75 (Cronbach’s α).

*Body Mass Index* of the patients was attained using the Tanita Multi-Frequency Body Composition Analyzer MC-180MA (Tanita Corporation, Tokyo, Japan). This device is repeatedly revised to meet the reference standards dual-energy X-ray absorptiometry^1^, and has been validated against other weighing methods (Strain et al., 2008). A stadiometer was used to measure height.

The Supplementary Table 1 includes the distribution of the measures in the study (direct means and standard deviations) stratified according to diagnostic subtype, and the internal consistency in the sample (Cronbach’s α coefficients). The raw scores (original data) of the questionnaires (CET, EDI-2, and SCL-90-R) was analyzed.

### Procedure

Psychologists and psychiatrists (all extensively trained in the use of the instruments) conducted clinical and physical evaluations during two clinical structured face-to-face interviews. Assessment instruments were also administered upon first evaluation. The controls (students from the University of Barcelona) completed the necessary printed questionnaires throughout the academic year 2014–2015. Self-reported height and weight were provided alongside the questionnaires. Written informed consent was obtained from all participants prior to their inclusion in the study, and the Ethics Committee of the University Hospital of Bellvitge approved the study. The study was conducted in accordance with the Declaration of Helsinki.

### Statistical Analysis

Analyses were carried out with SPSS20 and Mplus7.4 for Windows. First, confirmatory factor analysis was used to assess the validity of the original CET construct model (based on the five dimensions: avoidance and rule-driven behavior, weight control exercise, mood improvement, lack of enjoyment, and exercise rigidity) for the groups studied. Goodness-of-fit was examined for (Kline, 2010): RMSEA < 0.10, CFI > 0.90, Tucker-Lewis Index TLI > 0.90, and SRMR < 0.10. Next, analysis of variance (ANOVA) adjusted by the participants’ sex and age was conducted to compare clinical measures between the groups studied (ED diagnostic subtypes and controls). Finally, partial correlations adjusted by participants’ sex and age were used to analyze the association between CET scores and ED and general psychopathology scores (EDI-2 scales and SCL-90R scales, respectively).

In this study, results were considered statistically significant at a significance threshold of *p* ≤ 0.05. In addition, *p*-values depend on both the magnitude of associations and the precision of the estimate; effects of small magnitude that may be clinically unimportant can seem “significant” when the sample size is large and the opposite can occur whereby a large effect size could emerge from a small sample without it reaching significance. Therefore, both the significance test and estimation of effect sizes were taken into account. Cohen’s-*d* coefficients were used to assess the strength of the associations in the ANOVA procedures (moderate effect size was considered for |*d*| > 0.50 and large for |*d*| > 0.80) and partial correlations were considered moderate if |*r*| > 0.24 and large if |*r*| > 0.30.

## Results

### Psychometrical Properties of the CET Spanish Version

Confirmatory factor analysis generated adequate goodness-of-fit for the five dimension structure (RMSEA = 0.087, CFI = 0.910, TLI = 0.900, and SRMR = 0.080), with Cronbach’s-α values for the CET dimensions ranging between α = 0.79 (exercise rigidity scale) and α = 0.96 (avoidance and rule-driven scale). Correlations between factors were high, ranging between *r* = 0.46 (between the weight control and mood improvement scales) and *r* = 0.69 (between the weight control and avoidance and rule-driven scales).

The model including the participants’ gender as a group to assess the invariance of the internal structure by sex also displayed an adequate fit (RMSEA = 0.097, CFI = 0.900, TLI = 0.900, and SRMR = 0.090), and joint tests for parameter classes confirmed invariance by measurement coefficients (χ^2^ = 16.95 and *p* = 0.458), covariance for measurement errors (χ^2^ = 28.64 and *p* = 0.192) and covariance for exogenous variables (χ^2^ = 7.98 and *p* = 0.158).

The Supplementary Table 2 includes the complete results from the confirmatory factor analysis.

### Comparison of CET and Clinical Measures across Diagnostic Subtypes

**Table 1** includes the results of the ANOVA, adjusted according to the covariates participants’ sex and age, comparing the CET and other clinical measures between the groups evaluated. The first part of the table presents the descriptive data for each group (the raw mean scores and standard deviations obtained from the questionnaires adjusted by the covariates sex and age), followed by the global *F*-test exploring the presence of overall differences between the groups, and finally the pairwise comparisons estimating mean differences between the ED diagnostic subtypes. Based on the test of significance from pairwise comparisons (*p*-value), the BN and EDNOS groups obtained higher mean scores compared to the control participants in the avoidance and rule-driven behavior, weight control exercise and total CET scales, and higher mean scores than the AN group across all scales except for the exercise rigidity scale. There were no differences between the AN patients and the control group in any of the CET scales, except for the mood improvement scale where the control participants scored higher. CET scores were statistically equal between the BN and EDNOS groups. Differences between groups were found in the mean EDI-2 (ED symptoms) total score in all pairwise comparisons, being highest in the BN patients, followed by the EDNOS patients, the AN patients and finally the control participants. A similar pattern was observed in terms of the SCL-90R (general psychopathology) GSI mean score, although in this case there were no differences between the AN patients and the EDNOS patients. Regarding the effect size for the pairwise comparisons, overall the contrasts that reached significance (*p* ≤ 0.05) were also of moderate to good effect size (*|d| >* 0.50). The exceptions were: AN vs. controls for mood improvement scale, EDNOS vs. controls for CET total, AN vs. BN for all the CET scales except for total score, AN vs. EDNOS for weight control exercise and CET total and BN vs. EDNOS for GSI scale.

**Table 1:** Comparison of CET raw scores and clinical measures between groups: ANOVA adjusted by participants’ sex and age.

											Pairwise comparisons: *p-value* and Cohen’s-*d*											
	Controls (*n* = 128)		AN (*n* = 40)		BN (*n* = 56)		EDNOS (*n* = 62)		Factor group		Controls–AN		Controls–BN		Controls–EDNOS		AN vs. BN		AN–EDNOS		BN–EDNOS	
	Mean	*SD*	Mean	*SD*	Mean	*SD*	Mean	*SD*	*F*_(3,283)_	*p*	*P*	*|d|*	*p*	*|d|*	*p*	*|d|*	*p*	*|d|*	*p*	*|d|*	*p*	*|d|*
Avoidance and rule-driven behavior	1.28	1.07	1.69	1.55	2.27	1.50	2.12	1.65	7.48	**<0.001^∗^**	0.128	0.30	**<0.001^∗^**	**0.76^†^**	**<0.001**	**0.60^†^**	**0.038^∗^**	0.39	0.124	0.27	0.538	0.10
Weight control exercise	2.15	1.28	2.54	1.46	3.36	1.37	3.08	1.49	10.97	**<0.001^∗^**	0.142	0.28	**<0.001^∗^**	**0.91^†^**	**<0.001**	**0.67^†^**	**0.003^∗^**	**0.58^†^**	**0.046^∗^**	0.37	0.266	0.20
Mood improvement	3.47	1.04	2.97	1.65	3.53	1.29	3.46	1.45	1.87	0.135	**0.042^∗^**	0.37	0.810	0.05	0.944	0.01	**0.034^∗^**	0.38	0.060	0.32	0.771	0.05
Lack enjoyment	2.66	1.00	2.31	1.32	2.87	1.29	2.91	1.31	2.60	0.053	0.131	0.29	0.314	0.19	0.178	0.22	**0.022^∗^**	0.43	**0.013^∗^**	**0.50^†^**	0.862	0.03
Exercise rigidity	2.38	1.29	2.05	1.54	2.56	1.55	2.44	1.51	1.08	0.359	0.229	0.24	0.480	0.13	0.807	0.04	0.081	0.33	0.181	0.26	0.639	0.08
Total CET score	10.79	3.79	10.75	5.52	13.66	4.71	12.85	5.31	6.18	**<0.001^∗^**	0.956	0.01	**0.001^∗^**	**0.67^†^**	**0.005^∗^**	0.45	**0.002^∗^**	**0.57^†^**	**0.024^∗^**	0.39	0.350	0.16
EDI-2: Total score	37.4	26.0	80.5	43.4	122.4	38.0	100.0	45.5	68.90	**<0.001^∗^**	**<0.001^∗^**	**1.21^†^**	**<0.001^∗^**	**2.61^†^**	**<0.001^∗^**	**1.69^†^**	**<0.001^∗^**	**1.03^†^**	**0.009^∗^**	0.44	**0.001^∗^**	**0.53^†^**
SCL-90R: Global Severity Index (GSI) score	0.8	0.5	1.6	0.8	1.9	0.7	1.7	0.7	48.42	**<0.001^∗^**	**<0.001^∗^**	**1.25^†^**	**<.001^∗^**	**2.02^†^**	**<0.001^∗^**	**1.53^†^**	**0.003^∗^**	**0.53^†^**	0.257	0.19	**0.038^∗^**	0.35

*AN, anorexia; BN, bulimia; EDNOS, eating disorder non-otherwise-specified; and SD, standard deviation.*

*^∗^Bold: significant result.*

*^†^Bold: moderate (|d| > 0.50) to good (|d| > 0.80) effect size.*

### Association between CET and Clinical Measures in the ED Patients

**Table 2** displays the partial correlations (adjusted by the covariates sex and age) between the CET scales and clinical profile of the ED sample (*n* = 158). The CET scales mood improvement and lack of enjoyment only obtained poor correlations with the EDI-2 and the SCL-90R scores, and the CET exercise rigidity scale only positively correlated with the EDI-2 drive for thinness scale. The strongest associations were found between the scales avoidance and rule-driven behavior, weight control exercise and total CET, and: (1) the EDI-2 scales drive for thinness, body dissatisfaction, interceptive awareness, asceticism, social insecurity and total EDI-2; and (2) the SCL-90R scales interpersonal sensitivity, depressive symptoms and anxiety and the global GSI and PST indexes.

**Table 2:** Partial correlations (adjusted by participants’ sex and age) between CET raw scores and clinical measures in the ED sample (*n* = 158).

CET scales →	Avoidance rule-driven behavior	Weight control exercise	Mood improvement	Lack enjoyment	Exercise rigidity	Total CET score
EDI: Drive for thinness	**0.408**^†^	**0.578**^†^	0.214	0.228	**0.300^†^**	**0.487**^†^
EDI: Body dissatisfaction	**0.277**^†^	**0.399**^†^	0.026	0.121	0.132	**0.301**^†^
EDI: Interoceptive awareness	**0.312**^†^	**0.342**^†^	0.071	0.108	0.125	**0.284**^†^
EDI: Bulimia	0.074	0.175	-0.012	0.074	-0.002	0.113
EDI: Interpersonal distrust	0.197	0.194	0.058	-0.005	0.031	0.136
EDI: Ineffectiveness	0.226	0.203	-0.010	0.015	0.070	0.174
EDI: Maturity fears	0.084	0.112	-0.044	0.023	0.009	0.038
EDI: Perfectionism	0.133	0.171	0.039	0.082	0.139	0.174
EDI: Impulse regulation	0.176	0.220	0.010	0.029	0.085	0.165
EDI: Asceticism	0.237	**0.355**^†^	-0.017	0.095	0.082	**0.248**^†^
EDI: Social insecurity	**0.248**^†^	0.199	0.012	-0.002	0.081	0.179
EDI: Total score	**0.325**^†^	**0.404**^†^	0.048	0.107	0.145	**0.316**^†^
SCL-90: Somatization	0.181	0.136	-0.002	0.042	0.065	0.131
SCL-90: Obsessive/compulsive	0.212	0.125	-0.011	0.077	0.061	0.163
SCL-90: Interpersonal sensitivity	**0.306**^†^	**0.275**^†^	0.065	0.127	0.137	**0.279**^†^
SCL-90: Depressive	**0.267**^†^	0.128	0.041	0.083	0.115	0.192
SCL-90: Anxiety	**0.243**^†^	0.129	0.037	0.084	0.042	0.152
SCL-90: Hostility	0.151	0.094	-0.006	0.001	0.078	0.111
SCL-90: Phobic anxiety	0.227	0.151	-0.067	-0.036	-0.050	0.116
SCL-90: Paranoid ideation	0.184	**0.248**^†^	-0.029	0.171	0.104	0.203
SCL-90: Psychotic	0.218	0.172	0.036	0.145	0.105	0.171
SCL-90: GSI score	**0.279**^†^	0.192	0.029	0.101	0.097	0.211
SCL-90: positive symptom total (PST) score	**0.279**^†^	0.204	0.042	0.169	0.076	0.218
SCL-90: Positive Symptom Distress Index (PSDI) score	0.239	0.156	0.066	0.045	0.144	0.200

*^†^Bold: effect size in the moderate (|r| > 0.24) to good range (|r| > 0.30).*

## Discussion

The present study assessed the validity of a Spanish version of the CET, and reviewed the potential differences between ED diagnostic subtypes and a healthy comparison group in terms of the dimensions of compulsive exercise as defined by the CET. Associations between these dimensions and ED symptoms and general psychopathology were also examined.

After being translated into Spanish, the initial five factor structure of the CET was preserved. In line with the proposed multidimensional conceptualization of compulsive exercise (Meyer and Taranis, 2011; Meyer et al., 2011), this study demonstrates that compulsive exercise may be incited by multiple factors. It also shows that the instrument is able to successfully distinguish individuals with a diagnosis of an ED from a non-clinical group on four of the five subscales and the global score of the CET.

Adding to the results obtained by Meyer et al. (2016), the findings in this study also show that exercise can be particularly compulsive in individuals with BN and EDNOS; the BN and EDNOS groups scored higher in the total CET scale compared to the healthy controls. These patients were more likely to engage in exercise because of body weight/shape concerns than the healthy controls. The CET scores in our study also indicate that the drive to exercise compulsively might emerge from the need to follow strict exercise rules and the fear that the terminating the exercise pattern could result in a negative emotional state and/or feelings of guilt (avoidance and rule-driven behavior scale). Moreover, continued exercise driven by the need to follow a strict exercise schedule seems to be of equal importance across all ED diagnostic subtypes and healthy controls.

Interestingly, AN patients did not differ from healthy controls in overall (global) compulsive exercise as defined by Meyer and Taranis (2011) and Meyer et al. (2011). The only discrepancy was in exercising for mood improvement, where scores were lower in the AN group. In addition, the patients with AN scored lower across all subscales compared to the patients with BN (apart from exercise rigidity), and exercise driven by weight/shape control and exercising despite a lack of enjoyment was less prevalent among these patients compared to those with EDNOS. Previous studies have shown excessive exercise to be particularly high in only a subgroup of individuals with AN (Davis et al., 1997; Shroff et al., 2006; Bewell-Weiss and Carter, 2010; Sauchelli et al., 2015). Bratland-Sanda et al. (2010) also found that patients with AN underreported the amount of exercise they engaged in. One of the explanations proposed by the authors is that individuals with AN have a different conceptualization of exercise, which could be reflected in the low CET scores observed in this study when compared to the other ED subtypes (Bratland-Sanda et al., 2010). Furthermore, as the disorder progresses, the physiological deterioration associated with it may be impeding the patients from engaging in exercise, which then becomes a secondary factor and receives less attention. Duration of the AN disorder has been found to be inversely linked to physical activity (Sauchelli et al., 2015). This was not evaluated in the present study, but it would be interesting to further assess the influence of this variable. Nonetheless, it has been claimed that the compulsive cognitions underlying the motivation to exercise should receive considerable attention from clinicians (Meyer and Taranis, 2011; Meyer et al., 2011).

The use of exercise for the control of body weight/shape in EDs is considered as a fundamental feature of EDs (Dalle Grave et al., 2008; Hechler et al., 2008). Accordingly, the ED participants in our study with more compulsive exercise-related cognitions showed greater body dissatisfaction and drive for thinness. An association was also found between poor interpersonal sensitivity and compulsive exercise, specifically exercise to control body weight/shape and avoidance and rule-driven exercise behavior. Individuals with EDs show a heightened sensitivity to the opinions and expectancies of others, often assume negative judgments and report negative social interactions (Atlas, 2004). In EDs, this is particularly strong in relation to one’s own appearance (Atlas, 2004). Therefore, individuals with EDs may exercise to control body weight and shape, which consequentially improves their self-esteem. When the individual believes that having an “ideal body” is the only way to achieve and maintain social approval, exercise might become ritualistic and compulsive; the fear of perceived criticism from others on appearance may result in an impulsive urge to exercise. In support, a recent study of college women has shown that body and eating-related social comparisons were directly associated with greater exercise-related thoughts and prolonged exercise behavior (Fitzsimmons-Craft et al., 2016).

Studies have reported continuous exercise in AN to serve the purpose of affect regulation (Peñas-Lledó et al., 2002; Bratland-Sanda et al., 2010). This study, parallel to that of Meyer et al. (2016); however, shows that exercise for mood improvement might be more important for healthy individuals than individuals with AN. A possible hypothesis is that, contrary to healthy individuals, emotion regulation in AN is primarily achieved by other factors inherent of the pathology. For example, it has been found that starvation-induced weight loss in acute AN patients is associated with an amelioration in the ability to regulate negative emotions (Brockmeyer et al., 2012, 2013). Therefore, in comparison to healthy individuals, AN patients might rely mostly on alternative strategies, such as starvation, to improve mood. Exercise may serve the function of partially inhibiting a negative state and not as much to induce positive feelings (Boyd et al., 2007; Vansteelandt et al., 2007).

Furthermore, in the present study compulsive exercise was predominant in the BN diagnostic category, but the bulimia subscale of the EDI-2 did not correlate with any of the CET subscales. This may be relevant when examining the origin of the pathological cognitions toward exercise. The EDI-2 subscale focuses primarily on the binge/purge symptoms of BN, such as eating when upset, eating/drinking in secret, or thinking about eating/binging/vomiting. It does not examine other facets of the disorder. One of these may be temperament. Cloninger (1987, 1999) described a psychobiological model that defines personality from a psychobiological and multi-dimensional perspective. It comprises four temperament traits, which have an inherited component and remain relatively stable across the lifespan, and three character traits, which are more modifiable in response to the environment. Individuals with BN show particularly high novelty-seeking in comparison to individuals with other ED and healthy controls (Atiye et al., 2015). This is a temperament trait characterized by both cognitive and behavioral impulsivity (Cloninger, 1999). Greater novelty-seeking has been associated with exercise behaviors in both non-clinical (Fernández-Aranda et al., 2014) and clinical (Dalle Grave et al., 2008) populations. It can therefore be hypothesized that the cognitive urge to exercise, as described by Taranis et al. (2011), is not merely associated with psychopathology, but temperament may also be an important vulnerability factor. The role of temperament was not examined in the current study, but should be explored in more depth in future studies.

There are several strengths to the current study. Most importantly, this study is the first to validate a Spanish translation of the CET, thus providing the groundwork for further research into this multi-faceted perspective of compulsive exercise in both clinical and nonclinical Spanish-speaking samples. In addition, the use of a large sample and the inclusion of a healthy comparison group increases the generability of our findings, and the study compares ED diagnostic subtypes (AN, BN, and EDNOS) that display varying psychopathology, personality, and behavioral profiles. However, some limitations need to be considered. First, the assessment of compulsive exercise was made via the use of a participative measure, which is vulnerable to reporting bias. Future studies should consider the use of objective instruments to assess actual exercise (frequency, intensity, and duration) in order to evaluate whether the compulsion to exercise reported by patients is reflected in daily exercise patterns, how these might prospectively influence psychopathology, and whether reducing physical activity may influence ED symptoms (or vice versa). Second, the clinical sample in this study comprised patients who were actively seeking treatment, and findings cannot be generalized to those individuals with an ED who are not receiving clinical attention and who might display more/less compulsive exercising.

The current study highlights the need to acknowledge that compulsive exercise is not a unitary but a multidimensional construct. Furthermore, it seems that the greater the psychopathology, the more likely it is that the exercise behavior is compulsive; the BN group presented the worst psychopathology and higher mean scores in the CET. Compulsive exercise poses an obstacle for treatment progress (Solenberger, 2001; Carter et al., 2004; Dalle Grave et al., 2008). Many clinicians have therefore favored the prohibition of exercise during treatment; however, some studies have shown that supervised, moderate physical activity programs may be beneficial (Alberti et al., 2013; Kostrzewa et al., 2013; Sauchelli et al., 2015). The present study shows that compulsive exercise should not be addressed equally in all ED, and interventions incorporating physical activity programs should be adapted to the distinct ED diagnostic subtypes. For example, particular attention should be placed on the high levels of compulsivity that were observed in the BN and EDNOS patients. Furthermore, when examining the presence of compulsive exercise in EDs and throughout the intervention process, it is essential that professionals take psychopathology into account.

## Author Contributions

All authors designed the work and revised it for important intellectual content. The data was gathered by SS, ZA, SJ-M, FF-A and AP-G. RG conducted the statistical analysis. SS, JA, and FF-A drafted the study. All authors revised, commented on and approved the final manuscript and are accountable for all aspects of the work.

## Conflict of Interest Statement

The authors declare that the research was conducted in the absence of any commercial or financial relationships that could be construed as a potential conflict of interest.

## Footnotes

1 http://www.bl-biologica.es/tanita_tbf.htm

## Abbreviations

AN: anorexia nervosa
BN: bulimia nervosa
CET: Compulsive Exercise Test
CFI: Comparative Fit Index
ED: eating disorder
EDI-2: Eating Disorder Inventory-2
EDNOS: eating disorders not-otherwise-specified
RMSEA: root mean square error of approximation
SCL-90-R: Symptom Checklist-90 Revised
SRMR: standardized root mean square residual
