# Supplementary material for: Dimensions of Compulsive Exercise across Eating Disorder Diagnostic Subtypes and the Validation of the Spanish Version of the Compulsive Exercise Test
Source: Front Psychol. 2016 Nov 24;7:1852. doi: 10.3389/fpsyg.2016.01852 (PMC5121244; doi:10.3389/fpsyg.2016.01852)
Supplement: Supplementary file 1 [file Table_1.DOCX]

Supplementary Material

**Dimensions of compulsive exercise across eating disorder diagnostic subtypes and the validation of the Spanish version of the Compulsive Exercise Test.**

Sarah Sauchelli, Jon Arcelus, Roser Granero, Susana Jiménez-Murcia, Zaida Agüera, Fernando Fernandez-Aranda^*^

*** Correspondence:** Fernando Fernández-Aranda: [ffernandez@bellvitgehospital.cat](mailto:ffernandez@csub.scs.es;)

**1 Supplementary Table 1**

Distribution of the psychometrical measures in the study

|  |  | HC (*n*=128) | | AN (*n*=40) | | BN (*n*=56) | | EDNOS (*n*=62) | |
| --- | --- | --- | --- | --- | --- | --- | --- | --- | --- |
|  | α | Mean | SD | Mean | SD | Mean | SD | Mean | SD |
| Avoidance - rule/driven | .96 | 1.34 | 1.07 | 1.62 | 1.55 | 2.17 | 1.50 | 2.12 | 1.65 |
| Weight control | .85 | 2.23 | 1.28 | 2.44 | 1.46 | 3.22 | 1.37 | 3.10 | 1.49 |
| Mood improvement | .90 | 3.54 | 1.04 | 2.90 | 1.65 | 3.42 | 1.29 | 3.47 | 1.45 |
| Lack enjoyment | .82 | 2.80 | 1.00 | 2.18 | 1.32 | 2.67 | 1.29 | 2.89 | 1.31 |
| Exercise rigidity | .79 | 2.48 | 1.29 | 1.95 | 1.54 | 2.43 | 1.55 | 2.42 | 1.51 |
| Total score | .92 | 11.05 | 3.79 | 10.47 | 5.52 | 13.23 | 4.71 | 12.89 | 5.31 |
| EDI: Drive for thinness | .93 | 4.65 | 5.68 | 9.59 | 7.51 | 16.91 | 3.85 | 14.53 | 6.16 |
| EDI: Body dissatisfaction | .94 | 6.87 | 7.67 | 13.46 | 7.73 | 19.36 | 7.28 | 16.05 | 9.04 |
| EDI: Interpersonal awaraness | .86 | 2.89 | 3.33 | 8.21 | 6.63 | 13.88 | 6.53 | 11.73 | 6.85 |
| EDI: Bulimia | .85 | 1.06 | 2.05 | 2.54 | 3.07 | 11.07 | 4.60 | 5.24 | 5.28 |
| EDI: Intepersonal distrust | .80 | 2.74 | 3.00 | 4.85 | 4.13 | 5.80 | 5.05 | 5.50 | 4.65 |
| EDI: Ineffectiveness | .91 | 2.80 | 4.17 | 10.72 | 8.51 | 12.88 | 7.04 | 11.03 | 7.23 |
| EDI: Maturity fears | .80 | 4.94 | 3.81 | 6.90 | 5.74 | 9.63 | 6.19 | 8.21 | 5.80 |
| EDI: Perfectionism | .71 | 4.84 | 3.66 | 4.69 | 4.01 | 6.14 | 4.18 | 6.87 | 4.36 |
| EDI: Impulse regulation | .82 | 1.47 | 2.55 | 5.87 | 6.72 | 8.04 | 6.03 | 6.50 | 5.51 |
| EDI: Ascetism | .70 | 2.76 | 2.55 | 5.59 | 4.31 | 8.80 | 3.46 | 7.52 | 4.56 |
| EDI: Social insecurity | .82 | 2.93 | 3.16 | 7.31 | 5.16 | 8.46 | 5.40 | 7.44 | 4.89 |
| EDI: Total score | .97 | 37.96 | 26.02 | 79.72 | 43.43 | 120.96 | 38.00 | 100.61 | 45.49 |
| SCL-90: Somatization | .92 | 0.70 | 0.58 | 1.67 | 0.93 | 2.06 | 0.81 | 1.86 | 0.97 |
| SCL-90: Obsessive/compul. | .87 | 1.05 | 0.63 | 1.72 | 0.95 | 2.09 | 0.82 | 1.88 | 0.86 |
| SCL-90: Interponal sensit. | .89 | 0.96 | 0.69 | 1.73 | 0.96 | 2.26 | 0.88 | 2.05 | 0.95 |
| SCL-90: Depressive | .94 | 0.90 | 0.65 | 2.14 | 0.97 | 2.54 | 0.84 | 2.16 | 0.91 |
| SCL-90: Anxiety | .91 | 0.71 | 0.56 | 1.53 | 0.96 | 1.96 | 0.89 | 1.73 | 0.85 |
| SCL-90: Hostility | .87 | 0.59 | 0.58 | 1.20 | 1.01 | 1.38 | 1.04 | 1.28 | 0.87 |
| SCL-90: Phobic anxiety | .85 | 0.21 | 0.35 | 0.90 | 0.96 | 1.22 | 0.91 | 0.98 | 0.85 |
| SCL-90: Paranoid ideation | .78 | 0.83 | 0.62 | 1.23 | 0.85 | 1.57 | 0.88 | 1.42 | 0.81 |
| SCL-90: Psychotic | .86 | 0.43 | 0.46 | 1.15 | 0.75 | 1.50 | 0.78 | 1.22 | 0.74 |
| SCL-90: GSI score | .98 | 0.74 | 0.47 | 1.58 | 0.77 | 1.97 | 0.69 | 1.72 | 0.73 |
| SCL-90: PST score | .98 | 40.05 | 18.48 | 59.20 | 17.50 | 68.50 | 14.82 | 64.02 | 17.76 |
| SCL-90: PSDI score | .98 | 1.55 | 0.37 | 2.27 | 0.61 | 2.52 | 0.57 | 2.32 | 0.57 |

*Note.* HC: healthy controls. AN: anorexia nervosa. BN: bulimia nervosa. EDNOS: eating disorder non-otherwise-specified. EDI: Eating Disorder Inventory-2; SCL-90: Symptom Checklist Revised 90

α: Cronbach’s alpha in sample.
