# Supplementary material for: Dimensions of Compulsive Exercise across Eating Disorder Diagnostic Subtypes and the Validation of the Spanish Version of the Compulsive Exercise Test
Source: Front Psychol. 2016 Nov 24;7:1852. doi: 10.3389/fpsyg.2016.01852 (PMC5121244; doi:10.3389/fpsyg.2016.01852)
Supplement: Supplementary file 2 [file Table_2.DOCX]

Supplementary Material

**Dimensions of compulsive exercise across eating disorder diagnostic subtypes and the validation of the Spanish version of the Compulsive Exercise Test.**

Sarah Sauchelli, Jon Arcelus, Roser Granero, Susana Jiménez-Murcia, Zaida Agüera, Fernando Fernandez-Aranda^*^

*** Correspondence:** Fernando Fernández-Aranda: [ffernandez@bellvitgehospital.cat](mailto:ffernandez@csub.scs.es;)

**2 Supplementary Table 2**

Confirmatory Factor Analysis (includes invariance of parameters for groups defined by sex)

|  | Complete sample | | | | | Standardized coefficients including sex invariance | | | | | | Test of invariance | |
| --- | --- | --- | --- | --- | --- | --- | --- | --- | --- | --- | --- | --- | --- |
|  | Standardized coefficients | | | | | Women | | | Men | | | (measurem.coeff) | |
|  | B | SE | *p* | 95%CI (B) | | B | SE | *p* | B | SE | *p* | χ^2^*(df=1)* | *p* |
| F1. Avoidance and rule/driven |  |  |  |  |  |  |  |  |  |  |  |  |  |
| 9. Depressed | .856 | .024 | <.001 | 0.808 | 0.903 | .836 | .021 | <.001 | .829 | .039 | <.001 | 0.089 | 0.766 |
| 10. Guilty | .867 | .023 | <.001 | 0.823 | 0.911 | .879 | .016 | <.001 | .861 | .034 | <.001 | 0.063 | 0.801 |
| 11. Insistent | .714 | .043 | <.001 | 0.630 | 0.797 | .748 | .030 | <.001 | .627 | .057 | <.001 | 2.016 | 0.156 |
| 15. I don’t miss | .693 | .045 | <.001 | 0.605 | 0.782 | .729 | .031 | <.001 | .622 | .058 | <.001 | 1.562 | 0.211 |
| 16. Feel agitated | .919 | .015 | <.001 | 0.890 | 0.949 | .900 | .014 | <.001 | .907 | .026 | <.001 | 1.094 | 0.296 |
| 20. Angry-frustrated | .924 | .014 | <.001 | 0.896 | 0.952 | .917 | .012 | <.001 | .853 | .035 | <.001 | 0.083 | 0.773 |
| 22. Let down | .838 | .027 | <.001 | 0.786 | 0.890 | .860 | .018 | <.001 | .824 | .040 | <.001 | 0.064 | 0.801 |
| 23. Anxious | .917 | .015 | <.001 | 0.886 | 0.947 | .871 | .017 | <.001 | .816 | .041 | <.001 | 0.146 | 0.703 |
| F2. Weight control |  |  |  |  |  |  |  |  |  |  |  |  |  |
| 2. Appareance | .736 | .044 | <.001 | 0.650 | 0.821 | .817 | .025 | <.001 | .641 | .063 | <.001 | 2.566 | 0.109 |
| 6. More exercise | .866 | .026 | <.001 | 0.815 | 0.916 | .846 | .022 | <.001 | .789 | .056 | <.001 | 1.470 | 0.225 |
| 8. Not slim | .310 | .079 | <.001 | 0.154 | 0.465 | .377 | .057 | <.001 | .260 | .053 | <.001 | 0.263 | 0.608 |
| 13. Lose weight | .845 | .029 | <.001 | 0.789 | 0.902 | .820 | .025 | <.001 | .721 | .060 | <.001 | 0.533 | 0.466 |
| 18. Worry | .895 | .023 | <.001 | 0.850 | 0.940 | .851 | .022 | <.001 | .695 | .061 | <.001 | 0.399 | 0.528 |
| F3. Mood improvement |  |  |  |  |  |  |  |  |  |  |  |  |  |
| 1. Feel happy | .834 | .031 | <.001 | 0.774 | 0.895 | .820 | .024 | <.001 | .722 | .057 | <.001 | 0.514 | 0.473 |
| 4. Less anxious | .769 | .038 | <.001 | 0.694 | 0.844 | .753 | .031 | <.001 | .657 | .061 | <.001 | 2.037 | 0.154 |
| 14. Less tense | .875 | .026 | <.001 | 0.825 | 0.925 | .904 | .016 | <.001 | .820 | .049 | <.001 | 2.147 | 0.143 |
| 17. Improves mood | .850 | .028 | <.001 | 0.795 | 0.906 | .855 | .021 | <.001 | .730 | .057 | <.001 | 0.744 | 0.388 |
| 24. Less depressed | .694 | .047 | <.001 | 0.601 | 0.787 | .782 | .028 | <.001 | .545 | .061 | <.001 | 2.785 | 0.095 |
| F4. Lack of enjoyment |  |  |  |  |  |  |  |  |  |  |  |  |  |
| 5. Chore | .288 | .134 | .032 | 0.025 | 0.551 | .288 | .094 | .002 | .444 | .135 | .001 | 0.314 | 0.575 |
| 12. Enjoy | .321 | .144 | .025 | 0.040 | 0.603 | .365 | .110 | .001 | .486 | .142 | .001 | 0.229 | 0.632 |
| F5. Exercise rigidity |  |  |  |  |  |  |  |  |  |  |  |  |  |
| 3. Organization | .660 | .055 | <.001 | 0.553 | 0.768 | .630 | .043 | <.001 | .560 | .073 | <.001 | 2.494 | 0.114 |
| 7. Repetitive | .852 | .036 | <.001 | 0.782 | 0.922 | .866 | .026 | <.001 | .753 | .069 | <.001 | 0.464 | 0.496 |
| 19. Routine | .829 | .037 | <.001 | 0.755 | 0.902 | .805 | .030 | <.001 | .709 | .070 | <.001 | 0.878 | 0.349 |
| Fit statistics: RMSEA: .087; CFI: .91; TLI: .90; SRMR: .08 | | | | | | Fit statistics: RMSEA: .097; CFI: .90; TLI: .90; SRMR: .09 | | | | | | | |
| Correlations between factors | F1 | F2 | F3 | F4 | F5 | Joint tests for parameter classes | | | | χ^2^ | *df* | *p* |  |
| F1. Avoidance and rule/driven | - |  |  |  |  | Measurement coefficients | | | | 16.95 | 17 | .458 |  |
| F2. Weight control | .693 | - |  |  |  | Covariances measurement errors | | | | 28.64 | 23 | .192 |  |
| F3. Mood improvement | .584 | .460 | - |  |  | Covariances exogenous variables | | | | 7.977 | 5 | .158 |  |
| F4. Lack of enjoyment | .624 | .549 | .660 | - |  |  | | | |  |  |  |  |
| F5. Exercise rigidity | .597 | .544 | .530 | .625 | - |  | | | |  |  |  |  |
